# Formulation Development and Evaluation of Fast Disintegrating Tablets of Salbutamol Sulphate, Cetirizine Hydrochloride in Combined Pharmaceutical Dosage Form: A New Era in Novel Drug Delivery for Pediatrics and Geriatrics

**DOI:** 10.1155/2015/640529

**Published:** 2015-02-25

**Authors:** Deepak Sharma, Gurmeet Singh, Dinesh Kumar, Mankaran Singh

**Affiliations:** ^1^Department of Pharmaceutics, Rayat Bahra Institute of Pharmacy, Hoshiarpur, Punjab 146001, India; ^2^ISF College of Pharmacy, Ghal Kalan, Moga, Punjab 142001, India; ^3^Institute of Microbial Technology, Sector 39-A, Chandigarh 160036, India; ^4^Quantum Solutions, Chandigarh 160101, India

## Abstract

The objective of the present study was to prepare the fast disintegrating tablet of Salbutamol Sulphate, Cetirizine Hydrochloride in combined tablet dosage form for respiratory disorders such as bronchitis, asthma, and coughing for pediatrics and geriatrics. The tablets were prepared by direct compression technique. Superdisintegrant such as Sodium Starch Glycolate was optimized as 4% on the basis of least disintegration time. Different binders such as MCC and PVP K-30 were optimized along with optimized superdisintegrant concentration. 1% MCC was selected as optimum binder concentration on the basis of least disintegration time. The tablets were evaluated for hardness, friability, weight variation, wetting time, disintegration time, and drug content uniformity. Optimized formulation was further evaluated by in vitro dissolution test, drug-excipient compatibility, and accelerated stability study. Percent weight variation and content uniformity were within the acceptable limit. The friability was less than 1%. The wetting time and disintegration time were practically good for all formulations. FTIR studies and accelerated stability study showed that there was no interaction between the drug and excipients. It was concluded that, by employing commonly available pharmaceutical excipients such as superdisintegrants, hydrophilic and swellable excipients and proper filler, a fast disintegrating tablet of Salbutamol Sulphate, Cetirizine Hydrochloride in combined tablet dosage form, were formulated successfully with desired characteristics.

## 1. Introduction

Fast disintegrating tablets (FDTs) have received ever-increasing demand during the last decade, and the field has become a rapidly growing area in the pharmaceutical industry. Recent advancements in novel drug delivery system (NDDS) aim to enhance safety and efficacy of drug molecule by formulating a convenient dosage form for administration in order to achieve better patient compliance. One such approach is “fast disintegrating tablet.” Many patients find it difficult to swallow tablets and hard gelatin capsules that results in high incidence of noncompliance and ineffective therapy [1, 2]. Fast disintegrating drug delivery systems (FDDDS) are a new generation of formulations which combine the advantages of both liquid and conventional tablet formulations and, at the same time, offer added advantages over both traditional dosage forms. They provide the convenience of a tablet formulation and also allow the ease of swallowing provided by a liquid formulation [3]. The concept of fast dissolving/disintegrating tablets emerged from the desire to provide patients with more conventional means of taking their medication when drinking water is not available and in certain cases such as motion sickness, sudden episodes of allergic attacks, or coughing. Particularly these types of difficulty are faced by pediatric and geriatric patients [4]. Recent developments in technology have presented viable dosage alternatives for pediatric, geriatric, bedridden, nauseous, or noncompliant patients [5].

Recent developments in the technology have prompted scientists to develop FDTs with improved patient compliance and convenience. Upon introduction into the mouth, these tablets dissolve or disintegrate in the mouth in the absence of additional water for easy administration of active pharmaceutical ingredients. The popularity and usefulness of the formulation resulted in development of several FDT technologies. FDTs are solid unit dosage forms, which disintegrate or dissolve rapidly in the mouth without chewing and water [6]. Fast disintegrating tablets (FDTs) are also known as “fast dissolving,” “mouth dissolving,” “quick disintegrating,” “orally disintegrating,” “orodispersible,” “melt-in-mouth,” “quick dissolving,” “porous tablets” and “effervescent drug absorption system” [7]. Drug dissolution and absorption as well as onset of clinical effect and drug bioavailability may be significantly greater than those observed from conventional dosage forms. The amount of drug that is subjected to first pass metabolism is reduced as compared to standard tablet [8]. The target populations for these new fast dissolving/disintegrating dosage forms have generally been pediatric, geriatric, and bedridden or mentally disabled patients. Patients with diarrhea, persistent nausea, or vomiting, who are traveling or who have little or no access to water, are also good candidates for FDTs [9].

Formulations of the drugs chosen in combination for the treatment of asthmatic cough and other respiratory disorders are available in the market in conventional tablet and liquid dosage forms. Liquid dosage forms are having their own limitation from stability and dose measurement perspectives. Tablets to be swallowed are resisted by children patients and patient compliance is an issue with such dosage forms. Hence they do not comply with the prescription, which results in high incidence of noncompliance and ineffective therapy. Salbutamol sulphate is a *β*_2_ receptor agonist widely used as bronchodilator to relieve acute asthma. A fast dissolving tablet form would thus be advantageous, as salbutamol sulphate is water soluble and its preparation into a fast dissolving form would render it to dissolve rapidly and thereby result in rapid absorption without any lag time [10]. Cetirizine hydrochloride (CTZ) is an orally active white, crystalline water soluble drug and selective second generation nonsedative H1-receptor antagonist indicated in upper respiratory allergies, pollinosis, urticaria, and atopic dermatitis and is also used as adjuvant in seasonal asthma. Due to sore throat conditions, the patient experiences difficulty in swallowing a tablet type of dosage form [11]. Hence the present investigation was undertaken for preparation of fast disintegrating tablet of Salbutamol Sulphate, Cetirizine Hydrochloride in combined dosage form with an aim of improving/enhancing patient convenience and compliance, reducing the lag time, and providing faster onset of action to relieve the respiratory disorders immediately.

## 2. Materials and Methods

### 2.1. Materials

Salbutamol sulphate and cetirizine hydrochloride were obtained as gift samples from Trojan Pharma, Baddi, India. Microcrystalline Cellulose (Avicel PH-102) was received as gift sample from NB Entrepreneurs, Nagpur, India. Sodium Starch Glycolate (Primojel) and directly compressible mannitol (D-mannitol) were obtained from Qualikems Fine Chem Pvt. Ltd. Sodium Stearyl Fumarate was purchased from HiMedia. Sodium Saccharin was obtained from Loba Chemie, Mumbai, and talc from Nice Chemicals Private Limited, Hyderabad, India. PVP K-30 was obtained from HiMedia. All the chemicals and reagents used in research work were of analytical grade.

### 2.2. Methods

#### 2.2.1. Selection of Excipients and Optimization of Their Concentration

In the development of fast disintegrating tablets, the most important parameter that needs to be optimized is the disintegration time. Fast disintegrating tablets were prepared firstly using different excipients (binders and superdisintegrants) and then evaluated for various parameters like friability, hardness, and disintegration time to select the best combination for formulation of fast disintegrating tablets. The combination with the lowest disintegration time, optimum hardness, and friability was selected for further study.

#### 2.2.2. Optimization of Superdisintegrant Sodium Starch Glycolate (Primojel)

Total six formulations (F1–F6) were manufactured to study the effect of type and concentration of superdisintegrants shown in Table 1. Tablets were manufactured by the technique of direct compression. Required quantity of each ingredient was taken for each specified formulation. The superdisintegrant in (1%, 2%, 4%, 6%, 8%, and 10%) concentration was used to develop the tablets. All the ingredients were passed through mesh number 60. All the ingredients were cogrind in a pestle motor. Finally talc and Sodium Stearyl Fumarate were added and mixed for 5 minutes. The mixed blend of excipients was compressed into tablets using 8 mm punch in multipunch tablet compression machine (Dhiman Industries, India).

#### 2.2.3. Optimization of Polyvinylpyrrolidone (PVP K-30) or Microcrystalline Cellulose (Avicel PH-102) as Binder along with Optimized Concentration of Superdisintegrant

Total 14 formulations (F1–F14) were manufactured to study the effect of type of binder with optimized concentration of superdisintegrants shown in Table 2. Tablets were manufactured by the technique of direct compression. Required quantity of each ingredient was taken for each specified formulation. Optimized concentration of Sodium Starch Glycolate along with different concentration of binders (PVP K-30, MCC) was used to develop the tablets. All the ingredients were passed through mesh number 60. All the ingredients were cogrind in a pestle motor. Finally talc and Sodium Stearyl Fumarate were added and mixed for 5 minutes. The mixed blend of excipients was compressed into tablets using 8 mm punch in multipunch tablet compression machine (Dhiman Industries, India).

#### 2.2.4. Final Formulation of Salbutamol Sulphate, Cetirizine Hydrochloride Fast Disintegrating Tablets

Fast disintegrating tablets of Salbutamol Sulphate, Cetirizine Hydrochloridein combined form were manufactured by direct compression technique, the formula of which is shown in Table 3. Required quantity of each ingredient was taken for specified formulation. Accurately weighed quantities of Salbutamol Sulphate, Cetirizine Hydrochloride taken with optimized concentration of superdisintegrant and binder with excipients were cogrind in geometric progression in a dry and clean mortar. All the ingredients were passed through mesh number 60. Finally talc and Sodium Stearyl Fumarate were added and mixed for 5 minutes. The mixed blend of excipients was compressed into tablets using 8 mm punch in multipunch tablet compression machine (Dhiman Industries, India).

### 2.3. Evaluation Parameters

#### 2.3.1. Weight Variation Test

Weight variation test was done by weighing 20 tablets individually by using digital weighting balance (Ohaus, USA). Calculate the average weight and compare the individual tablet weight to the average weight of 20 tablets (Table 4) [12].

#### 2.3.2. Thickness

The thickness of tablets was measured by placing tablet between two arms of the vernier caliper (Indian caliper industries, Ambala, India). Three tablets from each batch were taken and an average thickness was measured [12].

#### 2.3.3. Hardness

The strength of tablet is expressed as tensile strength (Kg/cm^2^). The tablet crushing load is the force required to break a tablet into halves by compression. It was measured using a Monsanto Hardness Tester (Perfit). Three tablets from each formulation batch were taken randomly and the average reading was noted [13].

#### 2.3.4. Friability

The friability of the tablets was measured in a Roche friabilator (Camp-bell Electronics, Mumbai). Tablets of a known weight (*W*_0_) or sample 10 tablets are dedusted in a drum for a fixed time (100 revolutions) and weighed (*W*) again. Percentage friability was calculated from the loss in weight as given in the equation as below. The weight loss should not be more than 1% [14]:

(1) Percentage  friability =Initial  weightW0−Final  weightWInitial  weightW0×100.

#### 2.3.5. In Vitro Disintegration Test

The disintegration time of the tablet was measured in water (37 ± 2°C) according to using Digital Tablet Disintegration Tester (Veego, India). The time in seconds taken for the complete disintegration of the tablet with no palpable mass in the apparatus was measured in seconds. Six tablets from each batch (formulation) were tested for the disintegration time calculations [15].

#### 2.3.6. Wetting Time

A piece of tissue paper folded twice was placed in a small Petri dish (ID6.5 cm) containing 6 mL of distilled water being taken. A tablet containing a small quantity of amaranth color was placed on this being put on the paper and the time for the upper surface of the tablet to become complete red was measured. Three trials for each were performed [16].

#### 2.3.7. Drug Content Uniformity

Ten tablets were powdered and the blend equivalent to 2 mg of salbutamol sulphate and 5 mg of cetirizine hydrochloride was weighed and dissolved in suitable quantity of 6.8 pH phosphate buffer. The solution was sonicated, filtered, and suitably diluted and the drug content was determined from simultaneous equation method by using Double Beam UV Spectrophotometer (UV-1800 Shimadzu) at 276 nm and 230 nm wavelengths corresponding to salbutamol sulphate and cetirizine hydrochloride, respectively. Each sample was analyzed in triplicate [17, 18].

#### 2.3.8. In Vitro Dissolution Study

In vitro dissolution studies for all the fabricated tablets were carried out using USP eight-stage dissolution testing apparatus-2 (paddle method) (Lab, India), at 50 rpm in 500 mL of phosphate buffer solution, pH 6.8 at 37 ± 0.5°C. 5 mL of aliquot was withdrawn at the specified time intervals, filtered through Whatman filter paper and assayed spectrophotometrically at 276 nm and 230 nm. An equal volume of fresh medium was replaced into the dissolution medium after each sampling, to maintain the constant volume throughout the test. Dissolution studies were performed in triplicate. Absorbance of these solutions was measured at their respective *λ*_max⁡_ using a Double Beam UV Spectrophotometer (UV-1800 Shimadzu) [17]. Cumulative percentage (%) drug release was calculated from simultaneous equation method which is given as

(2)  At  276 nm A1=0.0066Cs+0.0050Cc,

(3)  At  230 nm A2=0.023Cs+0.0338Cc,

where C_s_ is concentration of salbutamol sulphate and C_c_ is concentration of cetirizine hydrochloride.

By putting the values of absorbances *A*_1_ and *A*_2_ at their respective *λ*_max⁡_, the concentrations of salbutamol sulphate and cetirizine hydrochloride were obtained in sample solutions [18].

#### 2.3.9. Drug-Excipient Compatibility Studies

FTIR spectra of pure drugs and formulated FDT containing drugs were recorded on FTIR Spectrophotometer (Bruker, USA). This study generally includes FTIR spectroscopy and these are generally performed to confirm the drug-excipient compatibility. FTIR spectra of samples were recorded in scanning range of 4000 to 600 cm^−1^ and the resolution was 1 cm^−1^. FTIR scans were then evaluated for shifting and masking and appearance of new peaks due to drug-excipient incompatibility [19].

#### 2.3.10. Accelerated Stability Studies

Accelerated stability studies were performed out on formulated FDTs (formulated in three primary batches) which were wrapped in aluminium foil and then stored in air-tight containers that is impermeable to solid, liquid, and gases, for a period of one month as prescribed by ICH guidelines at temperature of 40 ± 2°C and at ambient humidity and at room temperature. The tablets were withdrawn on 15th, 30th day and analyzed for drug content, friability, hardness, and in vitro disintegration time [20].

## 3. Results and Discussion

An attempt was made in the present investigation to make a fast disintegrating tablet of Salbutamol Sulphate, Cetirizine Hydrochloride in combination by direct compression method by employing superdisintegrant such as Sodium Starch Glycolate and mannitol as directly compressible diluent and Sodium Saccharin was used to enhance palatability. MCC (Avicel PH 102) was used in the formulation as a disintegrant and a binder. To impart pleasant taste and improve mouth feel, Sodium Saccharin was included as sweetening agent which is 400 times sweeter than sucrose. Sodium Stearyl Fumarate was used as a lubricant because of its water soluble nature and directly compressible features.

### 3.1. Optimization of Superdisintegrant Sodium Starch Glycolate (Primojel)

For optimal bioavailability and rapid absorption, the selection of the optimum concentration of superdisintegrant is necessary for rapid disintegration of tablets. Superdisintegrant decreases the disintegration time, resulting in the enhancement of dissolution rate of the drug. Therefore, for the formulation of rapidly disintegrating dosage forms, the proper selection of optimum concentration of superdisintegrant is of vital importance in dosage form development of FDTs. The results for optimization of superdisintegrant concentration in FDTs by direct compression method are shown in Table 5.

From the evaluation parameters, it was observed that 4%* Sodium Starch Glycolate* was the optimum concentration for rapid tablet disintegration on the basis of the least disintegration time observed with F3 formulation. The superdisintegrant action of SSG is exhibited by swelling and capillary action which causes rapid disintegration of tablets. Due to its hydrophilic nature, it rapidly absorbs water and swells up to 200–300% of their own weight. It is used in concentration range of 4–8%. Disintegration time increases above 8% due to gelling effect of the SSG [21].

### 3.2. Optimization of Polyvinylpyrrolidone (PVP K-30) or Microcrystalline Cellulose (Avicel PH-102) as Binder along with Optimized Concentration of Superdisintegrant

To study the effect of binders with the optimized concentration of SSG on the disintegration time, hardness, and friability of tablets, total 14 formulations (F1–F14) were prepared using different concentration of polyvinylpyrrolidone (PVP K-30) or Microcrystalline Cellulose. The results for optimization of different binder in FDTs are given in Table 6.

It was observed from the evaluation parameters that the disintegration time of the formulation F8 was further decreased and friability and hardness of tablets comply with the IP limits. The least disintegration time was observed in* F8* formulation that is 1%* MCC* as compared to* F2* formulation that is 2%* PVP K-30*. The probable reason was that MCC has porous morphology, due to which water is “wicked” through the capillary action which causes the tablet to break apart rapidly. On the other hand, water soluble materials such as PVP K-30 dissolving faster rather than disintegrating. Therefore 1%* Microcrystalline Cellulose* was selected as optimum binder concentration selected for final formulation of Salbutamol Sulphate, Cetirizine Hydrochloride FDT.

### 3.3. Evaluation Parameters for Salbutamol Sulphate, Cetirizine Hydrochloride FDT

Tablets were prepared using direct compression technique. The drug content was found in the range of 85–115% of the label claim (acceptable limit) and friability of the tablets was found below 1% indicating a good mechanical resistance of tablets. The in vitro disintegration time (DT) of the tablets was found to be less than 60 sec as shown in Table 7. Since mechanical integrity is of paramount importance in successful formulation of FDTs, hence the hardness of tablets was determined. The wetting time was practically good for formulation. The formulated FDTs had shown low DT indicating suitability of formulation for mouth dissolving tablet.

### 3.4. In Vitro Dissolution Study

**Figure 1:**
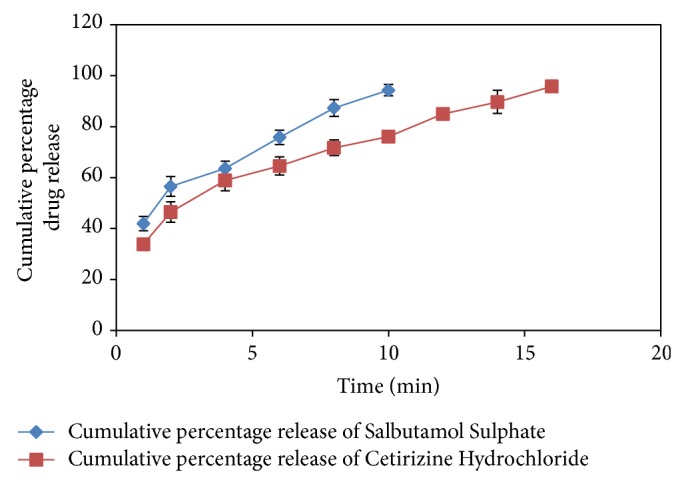
In vitro dissolution profile of Salbutamol Sulphate, Cetirizine Hydrochloride FDT.

From the in vitro dissolution data it was observed that 94.70 ± 2.24% of salbutamol sulphatereleased in 10 minutes and 95.59 ± 2.34% of cetirizine hydrochloride released in 16 minutes indicate that the tablet complies as per IP specifications that is 85%–110% shown in Figure 1.

### 3.5. Drug-Excipient Compatibility Studies

**Figure 2:**
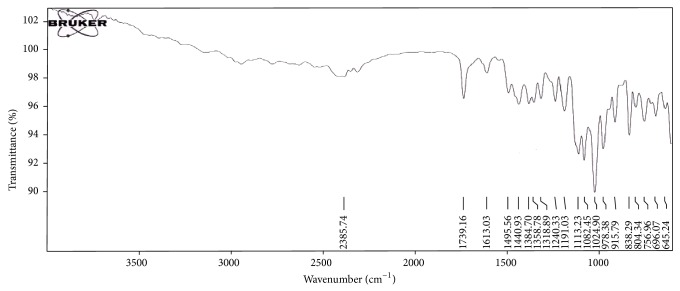
FTIR spectra of physical mixture of Salbutamol Sulphate, Cetirizine Hydrochloride.

**Figure 3:**
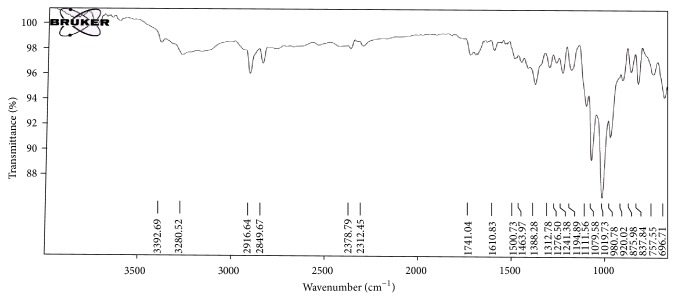
FTIR spectra of Salbutamol Sulphate, Cetirizine Hydrochloride FDT.

The results obtained with FTIIR studies showed that there was no interaction between the drug and other excipients used in the formulation. The FTIR spectra of Salbutamol Sulphate, Cetirizine Hydrochloride had shown intense absorption band at 1384.70 cm^−1^, 1613.03 cm^−1^, and 1384.70 cm^−1^ corresponding to the presence of functional groups such as Tri-methyl group, secondary amine group, and phenol group in salbutamol sulphate and at 756.96 cm^−1^, 1318.89 cm^−1^, 1024.90 cm^−1^, and 1191.03 cm^−1^ corresponding to the presence of functional groups such as aliphatic chloro compound, carboxylic acid, alkyl substituted ether, and tertiary amine in cetirizine hydrochloride. The FTIR of salbutamol sulphate, cetirizine hydrochloride FDT formulation showing intense absorption bands at 1388.28 cm^−1^, 1610.83 cm^−1^, and 1388.28 cm^−1^ and at 757.55 cm^−1^, 1312.78 cm^−1^, 1019.73 cm^−1^, and 1194.89 cm^−1^ indicates no change in the functional groups confirmed undisturbed structure of Salbutamol Sulphate, Cetirizine Hydrochloride, which indicates no drug-excipient incompatibility as shown in Figures 2 and 3.

### 3.6. Accelerated Stability Studies

Accelerated stability studies were carried out on formulated FDTs (formulated in three primary batches) as prescribed by ICH guidelines, wrapped in aluminium foil to prevent the formulation from exposure to light to simulate the aluminum packaging that is Alu Alu packing of drug products, and stored in air-tight containers which is impermeable to solid, liquid, and gases, for one-month period. The product is exposed to normal and extreme condition of temperature and humidity. The stability data of formulation was given in Tables 8 and 9.

The result of the stability study indicated that there were not much differences observed in hardness, disintegration time, drug content uniformity, and friability before and after the storage period at room temperature and at ambient humidity but at temperature of 40°C ± 2°C and at ambient humidity hardness was increasing with time, prolonging the DT of the tablet [22]; the probable reason was loss of moisture from tablets but in all cases DT is within the specified IP limit (within 3 min.). This indicates that formulation is fairly stable at both storage conditions.

## 4. Conclusion

Fast disintegrating tablets of Salbutamol Sulphate, Cetirizine Hydrochloride in combination were prepared by direct compression method using Sodium Starch Glycolate as a superdisintegrant and commonly available excipient. The tablets disintegrated rapidly in oral cavity and had acceptable hardness and friability. In vitro drug release from the tablets shows rapid drug dissolution. From the above study, it was concluded that, by employing commonly available pharmaceutical excipients such as superdisintegrants, hydrophilic and swellable excipients and proper filler, a fast disintegrating tablet of Salbutamol Sulphate, Cetirizine Hydrochloride FDT used in respiratory disorders, were formulated successfully with desired characteristics in combined pharmaceutical dosage form which disintegrated rapidly, providing rapid onset of action and enhancing the patient convenience and compliance. The technique adopted was found to be economical and industrially feasible.

## Figures and Tables

**Table 1:** Formula for 1 tablet (200 mg) of different concentration of SSG (data in mg).

Serial number	Ingredients	F1	F2	F3	F4	F5	F6
1	Salbutamol Sulphate	2	2	2	2	2	2
2	Cetirizine Hydrochloride	5	5	5	5	5	5
3	Sodium Starch Glycolate	2 (1%)	4 (2%)	8 (4%)	12 (6%)	16 (8%)	20 (10%)
4	Polyvinylpyrrolidone K-30	4	4	4	4	4	4
5	Sodium Stearyl Fumarate	3	3	3	3	3	3
6	Talc	3	3	3	3	3	3
7	Sodium Saccharin	5	5	5	5	5	5
8	Mannitol	176	174	170	166	162	158

**Table 2:** Formula for 1 tablet (200 mg) for the optimization of PVP K-30 or MCC with optimized concentration of SSG.

Serial number	Ingredients	F1	F2	F3	F4	F5	F6	F7	F8	F9	F10	F11	F12	F13	F14
1	Salbutamol Sulphate	2	2	2	2	2	2	2	2	2	2	2	2	2	2
2	Cetirizine Hydrochloride	5	5	5	5	5	5	5	5	5	5	5	5	5	5
3	Sodium Starch Glycolate	8	8	8	8	8	8	8	8	8	8	8	8	8	8
4	Polyvinylpyrrolidone K-30	2	4	6	8	10	12	14	—	—	—	—	—	—	—
5	Microcrystalline Cellulose	—	—	—	—	—	—	—	2	4	6	8	10	12	14
6	Sodium Stearyl Fumarate	2	2	2	2	2	2	2	2	2	2	2	2	2	2
7	Talc	2	2	2	2	2	2	2	2	2	2	2	2	2	2
8	Sodium Saccharin	5	5	5	5	5	5	5	5	5	5	5	5	5	5
9	Mannitol	174	172	170	168	166	164	162	174	172	170	168	166	164	162

**Table 3:** Formula of albutamol Sulphate, Cetirizine Hydrochloride FDT (data in mg).

Serial number	Ingredients	Formula for 1 tablet (200 mg)	Formula for 200 tablets (200 mg)
1	Cetirizine Hydrochloride	5	1000
2	Salbutamol Sulphate	2	400
3	Sodium Starch Glycolate	8	1600
4	Microcrystalline Cellulose	2	400
5	Sodium Stearyl Fumarate	4	800
6	Talc	2	400
7	Sodium Saccharin	8	1600
8	Mint Flavor	8	1600
9	Mannitol	161	32200

**Table 4:** Weight variation limit as per IP.

Average weight of tablet	Deviation %
80 mg or less	±10
More than 80 mg but less than 250 mg	±7.5
250 mg or more	±5

**Table 5:** Evaluation parameters for the optimization of Sodium Starch Glycolate.

Serial number	Evaluation parameters	F1 (1%)	F2 (2%)	F3 (4%)	F4 (6%)	F5 (8%)	F6 (10%)
1	Weight variation (IP)	Passed	Passed	Passed	Passed	Passed	Passed
2	Friability (%)	0.8	0.8	0.1	0.3	0.1	0.1
3	Hardness^*^ (Kg/cm^2^) ± S.D	2.2 ± 0.57	1.6 ± 0.28	1.5 ± 0.28	1.5 ± 0.32	2.0 ± 0.57	1.8 ± 0.28
4	Disintegration time^**^ (Sec) ± S.D	80 ± 2.34	59 ± 6.67	**42 ± 3.56**	49 ± 6.38	78 ± 7.39	95 ± 6.97

^*^Average of three determinations.

^**^Average of six determinations.

**Table 6:** Evaluation parameters for the optimization of PVP K-30 or MCC with optimized concentration of SSG.

Formula number	Evaluation parameters			
	Weight variation (IP)	Friability (%)	Hardness^*^ (Kg/cm^2^) ± S.D	Disintegration time^**^ (Sec) ± S.D
F1	Passed	0.1	2.2 ± 0.28	60 ± 1.78
F2	Passed	0.2	1.8 ± 0.28	**47 ± 2.45**
F3	Passed	0.5	2.0 ± 0.00	69 ± 2.89
F4	Passed	0.3	3.2 ± 0.76	83 ± 2.40
F5	Passed	0.3	1.6 ± 0.50	90 ± 5.16
F6	Passed	0.8	2.5 ± 0.50	120 ± 5.77
F7	Passed	0.8	2.0 ± 0.00	145 ± 5.43
F8	Passed	0.1	1.5 ± 0.50	**38 ± 4.34**
F9	Passed	0.1	1.5 ± 0.28	47 ± 2.34
F10	Passed	0.2	1.5 ± 0.28	62 ± 3.10
F11	Passed	0.1	1.8 ± 0.28	75 ± 1.32
F12	Passed	0.1	1.5 ± 0.28	82 ± 2.08
F13	Passed	0.1	1.8 ± 0.28	94 ± 3.67
F14	Passed	0.1	1.8 ± 0.28	110 ± 2.78

^*^Average of three determinations.

^**^Average of six determinations.

**Table 7:** Evaluation parameters for Salbutamol Sulphate, Cetirizine Hydrochloride FDT.

Serial number	Evaluation parameters	Results
1	Weight variation (IP)	Passed
2	Thickness^*^ (mm) ± S.D	3.63 ± 0.06
3	Hardness^*^ (Kg/cm^2^) ± S.D	1.8 ± 0.29
4	Friability (%)	0.3
5	Disintegration time^**^ (sec) ± S.D	45 ± 2.34
6	Wetting time^*^ (sec) ± S.D	28 ± 1.53
7	Drug content uniformity^*^ (mg) ± S.D	SAL-100.8 ± 3.36, CET-104.7 ± 1.97

^*^Average of three determinations.

^**^Average of six determinations.

**Table 8:** Stability data of Salbutamol Sulphate, Cetirizine Hydrochloride FDT at room temperature and at ambient humidity.

Evaluation parameters	Time interval								
	Data of three primary batches on								
	0 day			15th day			30th day		
	B-1	B-2	B-3	B-1	B-2	B-3	B-1	B-2	B-3
Hardness^*^ (Kg/cm^2^) ± S.D	1.5 ± 0.29	1.8 ± 0.29	1.5 ± 0.29	1.5 ± 0.00	1.5 ± 0.00	1.7 ± 0.29	1.5 ± 0.00	1.5 ± 0.29	1.5 ± 0.29
Friability (%)	1	0.6	1	0.2	0.3	0.2	0.1	0.1	0.1
Drug content uniformity^*^ (mg) ± S.D	SAL-100.8 ± 3.36, CET-104.7 ± 1.97	SAL-95.6 ± 2.34, CET-95.4 ± 2.86	SAL-93.8 ± 1.24, CET-97.7 ± 3.97	SAL-99.5 ± 2.14, CET-103 ± 1.76	SAL-94.5 ± 2.67, CET-98.6 ± 2.07	SAL-94.8 ± 1.23, CET-93.4 ± 1.77	SAL-98.3 ± 1.98, CET-97.8 ± 2.97	SAL-95.4 ± 1.65, CET-97.7 ± 2.75	SAL-95.7 ± 3.63, CET-94.2 ± 2.43
Disintegration time^**^ (sec) ± S.D	39 ± 2.28	47 ± 1.80	42 ± 3.01	42 ± 3.97	50 ± 4.52	47 ± 1.66	46 ± 2.83	49 ± 2.52	48 ± 3.75

^*^Average of three determinations/batches.

^**^Average of six determinations/batches.

**Table 9:** Stability data of Salbutamol Sulphate, Cetirizine Hydrochloride FDT at temperature (40° ± 2°C) and at ambient humidity.

Evaluation parameters	Time interval								
	Data of three primary batches on								
	0 day			15th day			30th day		
	B-1	B-2	B-3	B-1	B-2	B-3	B-1	B-2	B-3
Hardness^*^ (Kg/cm^2^) ± S.D	1.5 ± 0.29	1.8 ± 0.29	1.5 ± 0.29	2.5 ± 0.00	2.2 ± 0.29	2.5 ± 0.00	2.5 ± 0.00	2.5 ± 0.29	3.2 ± 0.29
Friability (%)	1	0.6	1	0.1	0.2	0.9	0.6	0.5	0.1
Drug content uniformity^*^ (mg) ± S.D	SAL-100.8 ± 3.36, CET-104.7 ± 1.97	SAL-95.6 ± 2.34, CET-95.4 ± 2.86	SAL-93.8 ± 1.24, CET-97.7 ± 3.97	SAL-98.5 ± 2.14, CET-100 ± 1.78	SAL-99.4 ± 2.67, CET-96.3 ± 2.07	SAL-90.42 ± 3.64, CET-95.7 ± 2.78	SAL-92.8 ± 1.98, CET-95.5 ± 1.97	SAL-99 ± 1.65, CET-98.1 ± 1.97	SAL-97.6 ± 3.63, CET-94.2 ± 1.63
Disintegration time^**^ (sec) ± S.D	39 ± 2.28	47 ± 1.80	42 ± 3.01	49 ± 2.38	55 ± 3.08	51 ± 1.76	55 ± 2.09	61 ± 1.89	58 ± 2.96

^*^Average of three determinations/batches.

^**^Average of six determinations/batches.
